# A CRISPR/CAS9‐based strategy targets the personalized chimeric neosequence in fusion‐driven cancer genome for precision medicine

**DOI:** 10.1002/ctm2.355

**Published:** 2021-03-17

**Authors:** Wei Huang, Zhan‐Cheng Zeng, Wen‐Tao Wang, Yu‐Meng Sun, Yue‐Qin Chen, Xue‐Qun Luo, Ke Fang

**Affiliations:** ^1^ Key Laboratory of Gene Engineering of the Ministry of Education School of Life sciences Sun Yat‐sen University Guangzhou China; ^2^ The First Affiliated Hospital Sun Yat‐sen University Guangzhou China

Dear Editor,

Recurrent chromosomal rearrangements leading to the generation of oncogenic fusion genes drive the formation of more than 17% of tumors.^1^ With the development of genome editing approaches, new possibilities for directly targeting the genomic sequence of cancer cells have arisen. Remarkably, CRISPR/CAS9 nuclease‐based genome editing is a well‐suited tool to target cancer‐causing mutations,^2^, ^3^ including single nucleotide polymorphisms (SNPs) and short insertions/deletions (indels),^4^ as they can create new protospacer adjacent motif (PAM) sequences. The genomic breakpoints of fusion genes are more suitable for CRISPR/CAS9 targeting than SNPs and short indels due to the wide range of PAM appearance and the high tolerance of base mismatch (Figure 1A). Furthermore, in contrast to disease‐related exonic mutations,^4^, ^5^ the breakpoints of fusion genes often occur in intronic regions, which decrease the risk of mistargeting the coding sequences of allele genes. Based on these observations, we report a strategy to specifically and efficiently target cancer cells carrying fusion genes by designing fusion‐site single guide RNAs (fsgRNAs), which anchor the breakpoint sequence (named “chimeric neosequence”) of the fusion gene for each patient (Figure 1B).

**FIGURE 1:**
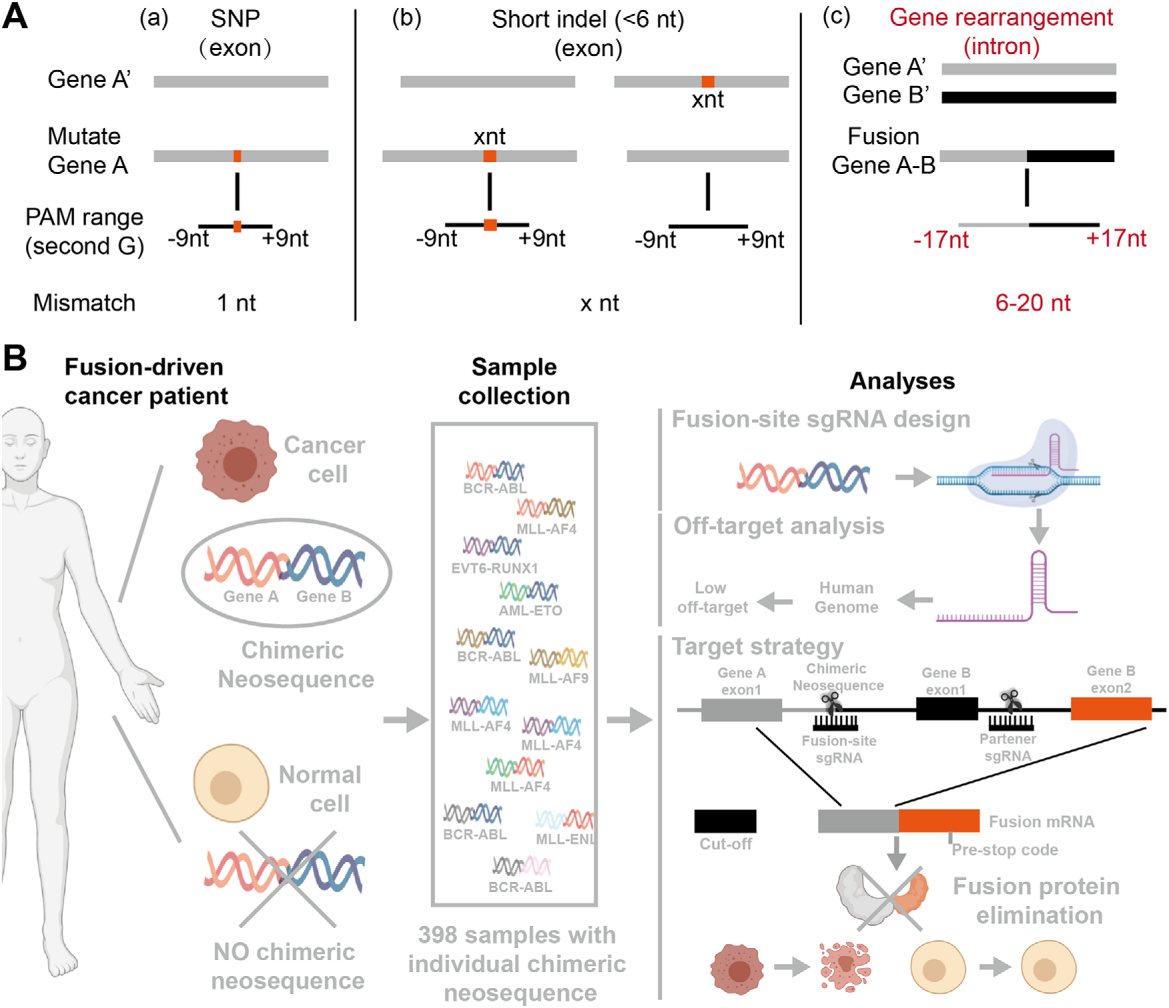
The workflow of chimeric neosequence targeting strategy. (A) A schematic diagram shows the PAM range and sgRNAs mismatch numbers in disease‐related mutations, SNPs, short indels, and gene rearrangements. The current targeting mutations, such as SNP and short indels (X nucleotides (nt) indel; X < 6 nt in 95% cases^5^), should occur within or near the 1‐8 nt PAM sequence (NGG; in case of spCAS9), and the mismatch bases compared with the allele gene are 1 nt and x nt. The chimeric neosequence of the fusion gene is well‐suited to be targeted by the system due to the wide range of PAM appearance (−17 to +17 bps from the fusion site; according to the previous report of the efficient sgRNAs length^6^) and due to the bases mismatch (6‐20 nt). (B) Workflow for chimeric neosequence analyses. A three‐step analysis (right) was established to study the feasibility of the targeting strategy: (1) design suitable fsgRNA to target each chimeric neosequence, (2) analyze the off‐target rate of fsgRNA, and (3) eliminate cancer‐driver fusion protein by combining fsgRNA and psgRNA

To test this approach, we first investigated the feasibility of designing fsgRNAs. A total of 398 intronic sequences of the six most common hematopoietic cancer‐driver fusion genes in clinical samples^1^ from multiple GEO datasets were collected for investigation (Table S1). Notably, none of these samples had the same chimeric sequence, indicating that the fusion gene breakpoint is highly specific for each patient. With the prerequisite of PAM range for spCAS9, we calculated the occurrence of the PAM (counted as “0,1,2”) next to the fusion site (−17 to +17 nucleotides from breakpoint) and clustered it in Figure 2A. Most of the samples contained more than one PAM. No preference for PAM appearance was observed at any position (Figure 2A, upper panel and Figure S1). The rate of NGG/NCC appearance is shown in Figure S2. Of the 398 samples, 323 (81%) had at least one PAM in the chimeric neosequence (Figure 2B), suggesting the common occurrence of fsgRNAs among the cancer‐driver fusion genes. Furthermore, 323 samples contained 842 eligible fsgRNAs, which is 2.6 fsgRNAs per sample on average (Figure 2B). We also found that different fusion genes showed preference for fsgRNA numbers, for example, 3.2 fsgRNAs for BCR‐ABL and 2.1 sgRNAs for MLL‐AF4. Altogether, 70.78% of the samples had more than two fsgRNAs (Figure S3), indicating the diversity of the fsgRNAs.

**FIGURE 2:**
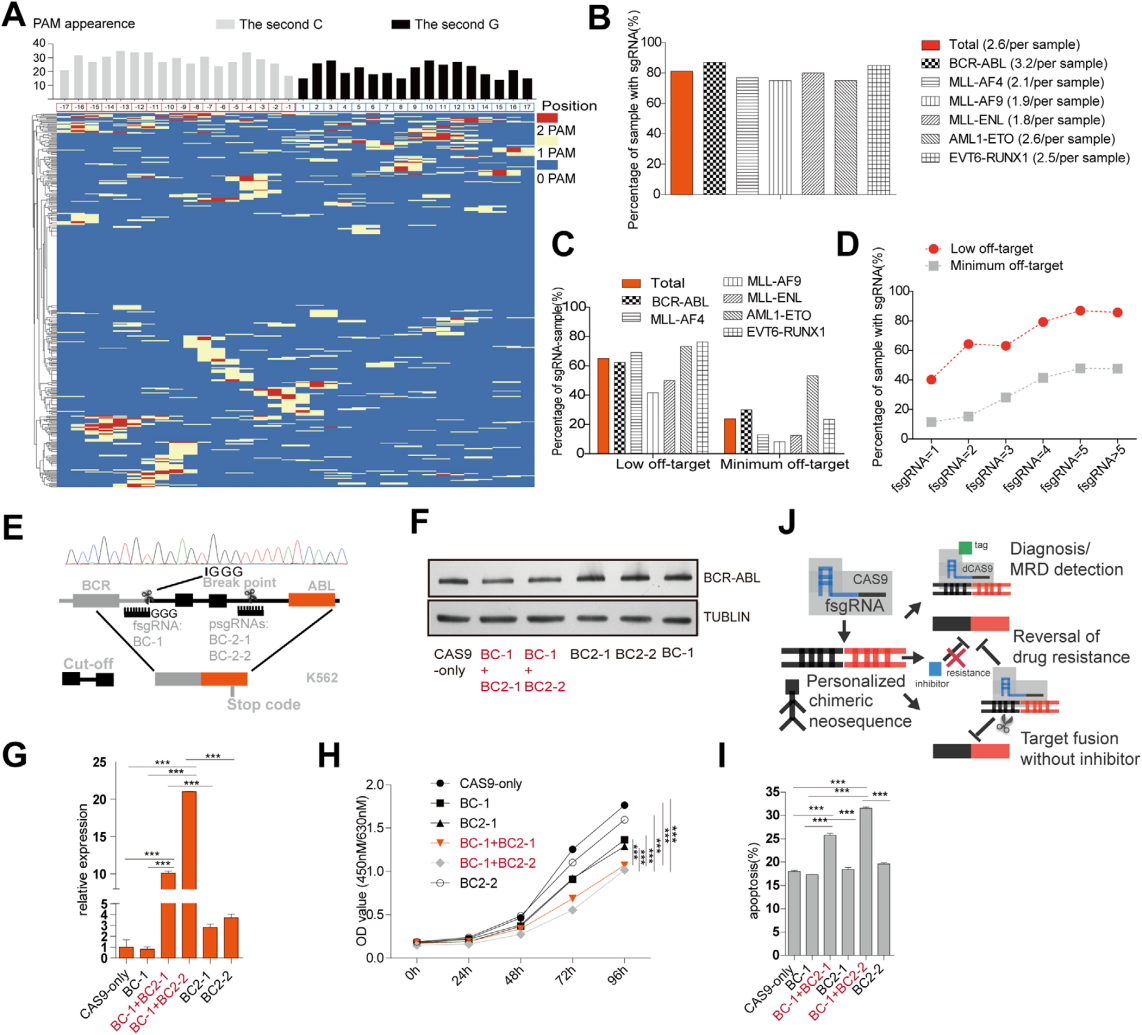
A CRISPR/CAS9‐based strategy targets the personalized chimeric neosequence to eliminate cancer‐driver fusion protein. (A) The candidate PAMs were detected within 17 bp from the two sides of the fusion‐site. The PAM in each position was counted (above). The occurrence of the PAM around the fusion‐site in 398 clinical samples is shown in the heatmap. If the position has no PAM sequence, it is indicated as 0, if there is one PAM it is indicated as 1, and if the position has two PAMs, it is indicated as 2 (bottom). (B) The fsgRNA rate showed that 81% (323/398) of the samples had at least one fsgRNA. The rate of each of the fusion proteins is presented. (C) The fsgRNAs were compared with the human genome, and the mismatches were counted. The percentages of the fsgRNAs with more than two mismatches in the seed sequence are shown in total and in six fusion genes. See also in Table S2. (D) The low off‐target rate of the samples was positively correlated with the number of fsgRNAs sites present in the samples. (E) A schematic diagram shows the strategy for deleting the fusion‐site exon of the BCR‐ABL locus using fsgRNA/psgRNA combination. (F) The BCR‐ABL protein levels were decreased in the fsgRNA/psgRNA complex samples. (G) qRT‐PCR assay showed the cutting‐off BCR‐ABL mRNA expression. Error bars reflect ±SEM (*, *p* < 0.05; **, *p* < 0.01; and ***, *p* < 0.001) in three independent experiments. (H, I) The fsgRNA/psgRNA complex (BC‐1+BC2‐1; BC‐1+BC2‐2) samples compared with the controls (CAS9‐only; BC‐1; BC‐2‐1; BC2‐2, respectively) induced lower proliferation (H) and higher apoptosis rate (I). Error bars reflect ±SEM (*, *p* < 0.05; **, *p* < 0.01; and ***, *p* < 0.001) in three independent experiments. (J) A schematic diagram shows the potential applications of targeting chimeric neosequences

We then examined the ability of fsgRNA to specifically target the oncogenic genes and to minimize unintended (“off‐target”) interactions. Importantly, no fsgRNA had another on‐target site in the human genome besides in the oncogenic gene, highlighting their specificity. Furthermore, Cas‐OFFinder^7^ and Off‐Spotter^8^ were used to calculate the potential off‐target sites of each fsgRNA based on two criteria^9^: (1) the fsgRNAs with more than two mismatches compared to the genome were chosen as low off‐target fsgRNAs; (2) the fsgRNAs with more than two mismatches within the seed sequence may have minimum off‐target effects. We found that 210 of 323 samples (65%) had at least one low off‐target fsgRNA, and 77 of 323 samples (24%) had at least one minimum off‐target fsgRNA (Figure 2C and Table S2). Notably, the higher fsgRNA number was positively correlated with the low and minimum off‐target rates (Figure 2D), suggesting that the diversity of the fsgRNAs could reduce the off‐target rate. The low off‐target rate shows the potential application of fsgRNAs in fusion‐driven cancer cell targeting.

Next, we designed a strategy to disrupt fusion gene translation using the CRISPR/CAS9 system (Figure 1B, right bottom). Briefly, two sgRNAs were specifically designed: the fsgRNA, which binds to the fusion gene loci, and the partner sgRNA (psgRNA), which targets the intron close to the fusion site. The binding of the psgRNA and fsgRNA results in the excision of the exon and the creation of a new open reading frame (ORF). When the fsgRNA and the psgRNA are designed, it is necessary to analyze the new ORF to guarantee that a premature termination codon is created as a result of the excision‐derived frameshifting. Thus, the combination of fsgRNA and psgRNA could eliminate fusion proteins and selectively kill cancer cells. The advantage of this approach is that no precise correction is required. Instead, imprecise deletions are sufficient to destroy fusion protein expression. We validated this strategy in the K562 cell line carrying the *BCR‐ABL* fusion gene. An fsgRNA named BC‐1 (Figure 2E) together with psgRNA, either named BC2‐1 or BC2‐2, was transfected into the cells. Compared to the controls (fsgRNA or psgRNAs with CRISPR/CAS9 protein, or the CRISPR/CAS9 protein only), transfecting the fsgRNA+psgRNA (BC‐1+BC2‐1; BC‐1+BC2‐2) with CRISPR/CAS9 protein decreased BCR‐ABL protein expression (Figure 2F) and increased the cutting‐off of the mRNA (Figure 2G). Further analyses showed that the fsgRNA/psgRNA combination reduced cell proliferation (Figure 2H) and induced apoptosis (Figure 2I and Figure S4). Collectively, these results indicate that the fsgRNA/psgRNA combination eliminates the fusion protein, which in turn affects cancer progression.

In conclusion, we provide a new strategy to link CRIPSR/CAS9‐based precision medicine to the intronic chimeric neosequence of cancer‐driver fusion genes. However, there are still several issues related to the use of CRISPR/CAS9 system in a clinical setup that remain to be addressed, such as the distinct editing efficiency in different cells.^3^ Thus, the development of tissue‐ and cell type‐specific delivery systems are required to enhance the efficacy of CRISPR/CAS9 system. Moreover, though it would seem uneconomical to design and test every fsgRNA for each patient, our strategy provides great potential in meeting the urgent and unmet medical need, including (Figure 2J): (1) diagnosis and detection of minimal residual disease by fsgRNA/dCAS9‐based gene loci imaging,^4^ (2) elimination of fusion proteins that are drug‐resistant (for instance, BCR‐ABL fusion protein resistant to tyrosine kinase inhibitors^10^), and (3) targeting those fusion genes without available inhibitors (e.g., MLL fusion gene, which causes MLL‐translocation‐driven leukemia1 with a low overall survival in classic treatment^1^). We expect that the future development of the CRISPR/CAS9 system can increase the feasibility of our strategy in fusion‐driven cancer diagnosis and treatment.

## CONFLICT OF INTEREST

The authors declare that there are no conflicts of interest.

## Supporting information

Supporting Information
