# Supplementary material for: A CRISPR/CAS9‐based strategy targets the personalized chimeric neosequence in fusion‐driven cancer genome for precision medicine
Source: Clin Transl Med. 2021 Mar 17;11(3):e355. doi: 10.1002/ctm2.355 (PMC7967915; doi:10.1002/ctm2.355)
Supplement: Supplementary file 1 — Supporting Information [file CTM2-11-e355-s001.docx]

**Title: A CRISPR/CAS9-based strategy targets the personalized chimeric neosequence in fusion-driven cancer genome for precision medicine.**

**Authors:** Wei Huang^1†^, Zhan-Cheng Zeng^1†^, Wen-Tao Wang^1^, Yu-Meng Sun^1^, Yue-Qin Chen^1^, Xue-Qun Luo^2*^, Ke Fang^1*^

**Materials and Methods**

Patient sample collection:

The patient samples were collected from the “nucleotide” in the NCBI database. The DNA breakpoint sequence was searched in UCSC and remarked as the different fusion partners. Each of the PAM was designed to the FsgRNA.

Plasmid construction:

The CRISPR plasmid PX458(GFP) and PX459 were cleavage by Bbsl1 and link the sgRNAs using T4 DNA ligase 10 min at room temperature. And the construction plasmid was used for Transfection. The sgRNAs sequence were: BC-1: GACCCTGGCCGCTGTGGAGT BC2-1: AAGAGAATCATACCAGTCCC BC2-2: GTGTTGATAGGTGGTCCCGA

Cell lines and cell cultures

K562 cells were cultured in RPMI 1640 (HyClone, SH30027) containing 10% fetal bovine serum (HyClone, SV30160) in a humidified atmosphere containing 5% CO2 at 37 °C.

RNA isolation and Quantitative real-time PCR

The total RNA was extracted from samples with TRIzol (Invitrogen, 15596) according to the manufacturer’s instructions. Real-time PCR was performed to quantify mRNA expression using ExTaq SYBR Green Supermix (Bio-Rad, Hercules, CA, USA) according to the manufacturer’s instructions. The qPCR primers of the cutting-off BCR-ABL mRNA was:

F: CGGGAGCAGCAGAAGAAGTGT R: TTCAGAAACCCATAGAGCCCC

Transfection

Cells were transfected using the Neon® Transfect ion System (Invitrogen) with 1ug plasmid in 10 μl reactions according to the manufacturer’s instructions.

Western blot

Protein extracts were boiled in RIPA buffer (Beyotime, P0013) and separated in a sodium dodecyl sulfate polyacrylamide electrophoresis (SDS-PAGE) gel. The proteins were then transferred to a polyvinylidene fluoride membrane (Millipore, HVPPEAC12) and probed with antibodies (anti-BCR-ABL, anti-TUBULIN and anti-PARP1 (Cell Signalling Technology, Boston, MA, USA)) After a 1-hincubation with anti-rabbit (Sigma-Aldrich) HRP-conjugated secondary antibody, the protein level was detected using a luminal reagent (Millipore). X-ray films were acquired and quantified with the densitometric software Quantity One (Bio-Rad).

Proliferation and apoptosis assays.

Cell proliferation was assessed using the Cell Counting Kit-8 (CCK-8). After transfection,2*10^4^ cells per well were plated in 96-well sterile plastic culture plates, and the CCK-8 assay (Dojindo Molecular Technologies,Shanghai, China) was performed after 0, 24, 48, 72, and 96 h. To assess the rate of apoptosis, transfected cells were harvested and washed twice with cold PBS, and the Annexin V-PI Kit (Nanjing Keygen, Nanjing, China) was used according to the manufacturer’sguidelines. The detection was performed with a FACS Calibur using CellQuest software (BDIS, San Jose, CA, USA).


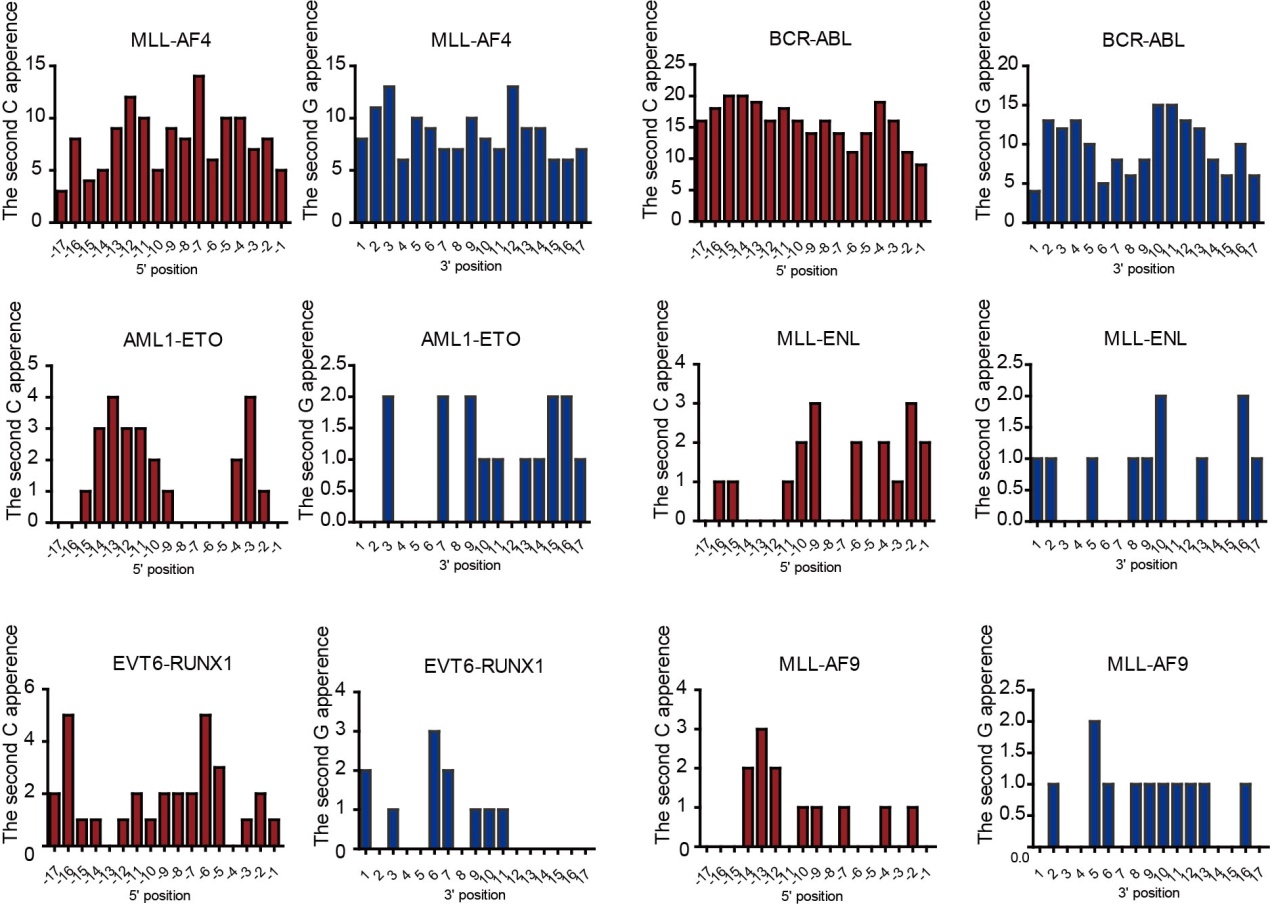


**Figure S1.** The appearances of NCC and NGG in six fusions were displayed according to the number of the second C or G in each position.


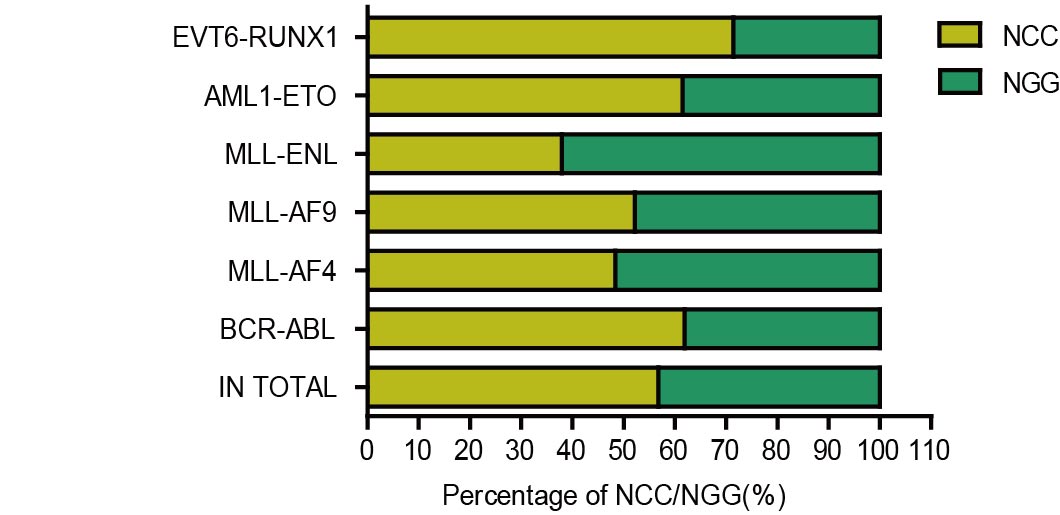


**Figure S2.** The NGG and NCC rate in total samples and in each fusion.


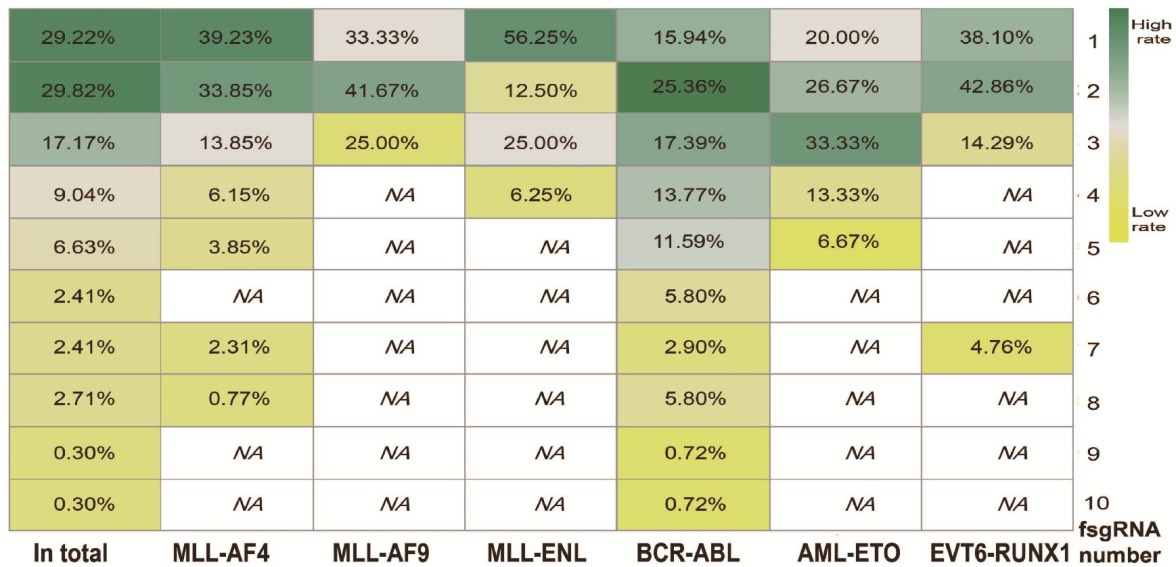


**Figure S3.** The rate of the sample containing different fsgRNA numbers, the fsgRNA numbers were counted from 1 to 10.


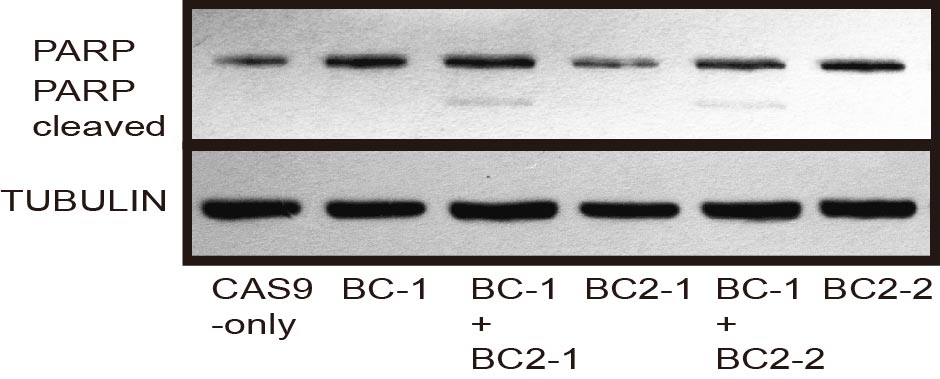


**Figure S4.** The western blot showed the apoptosis of fsgRNA/psgRNA complex that reflected by PARP1 cleavage.

**Table S1**. The chimeric neosequences of 398 leukemia samples with six common fusion proteins

| MLL-AF4 |  |
| --- | --- |
| TTTGTTACTTTCTATTTCCACT tggttcTTACCAAGTAAATAAA | isolate patient no 5049 |
| AAAGTATCAAACCATGATGATT ATATATTTACTTATTTTCTAAT | isolate patient no 4772 |
| GACCTTTCTCTCTCCACAGGAG TAGCTTAGGCCATTTGAAAGGA | isolate patient no 4597 |
| TACCAATTAATAAGAAAAAGGA gCAGGAGCTCTGGATTAAAGGC | isolate patient no 4415 |
| AGATGGGAGGCTTAGGAATCTT TTAACTTTGCCTACTTTCTTTG | isolate patient no 4006 |
| TTACTTTCTATTTCCACTGGTA ATACCCATGTTTCCATTGCTTG | isolate patient no 3402 |
| TTTCTATTTCCACTGGTATTAC TGCTGGAATAAGCCCAGGATAA | isolate patient no 4287 |
| TGTATATTTTTAAAAATCAAGG tgGATGGGAGAACAGAGGGGCA | isolate patient no 4279 |
| ACATGGAAAGTATCAAACCATG CACGATCACAGCTCACTTCAGC | isolate patient no 4705 |
| TTGTGATGTCACACTAATTTTA GAAGTGCATGAATTAAGAAAGG | isolate patient no 4654 |
| TTAAAGCAGCAGTTATTTccac TAGGTTCTGTATTCTATATAGA | isolate patient no 4424 |
| CTTTTTAAAGCAGCAGTTATTT CAAAAAAGTTCTTAcTTGCTAA | isolate patient no 4249 |
| ATGGAAAGTATCAAACCATGAT TGGAGTATTGTTAATCTTGTGG | isolate patient no 3729 |
| TCACTTGAGTCCAGGAGTTTAA TGACATTGTGATGTCACACTAA | isolate patient no 4793 |
| TGCTTTTCATCCTTATTTTCCA aaATGGAGGGAAAGGGAAGAAT | isolate patient no 4898 |
| TCAATAAGCAGGAGAATGCAGG TAAGTTCCTGGCGTCATAGGTA | isolate patient no 4868 |
| TTCCAGCAGATGGAGTCCACAG TCCCACTGAAAAAAAAAAAATT | isolate patient no 4777 |
| CTTTGGTCAGTGTTGTTAGGTC TTTCAGTCTCTTGAAGGGTGTG | isolate patient no 4278 |
| TTTCGCAATATATTCAATATGA GGCGGGTGGATCACCTGAGGTC | isolate patient no 4159 |
| ATGATGATTCCTTGAGTCAGCA cTTTTATCAGAGAAGAATTATA | isolate patient no 3479 |
| AAAAAAAAATTCAAAGATTATT CATCATGAAATGTCCTTAAAAA | isolate patient no 4697 |
| CTTCTATGTAGATGGCAGTGGA tcAAGACTATCCTTTTAATACT | isolate patient no 4093 |
| TATTAATAAAATTTGTCATTTG TGTTAGTCTTCACATGTGGCAG | isolate patient no 3524 |
| TATATTCATCTTTTGTCTCCTT GGCCTGAATTTAGGAAAAGGCA | isolate patient no 4850 |
| TTTCTCTGCCATTTCTCAGGGA GTACATATTTTTTTTGTACATG | isolate patient no 4787 |
| TGTATTCTATTTTGTAGGGAAA GATTGCCAGGAATCCAAACACA | isolate patient no 4704 |
| CCCTTCCCTGTATTCACTATTT atcAAAAAAAGTGAGTTGATCA | isolate patient no 4215 |
| CCGAGACTCCGTCTCAAAAAAA TGTAAAATAACTACACTGTGGT | isolate patient no 4194 |
| AAAGCAAGACCCAGTCTCTTTT tTACTAATTTTCTTTAATAAAG | isolate patient no 3812 |
| CCAGCCTGGGCAGCACAGTGAC GACAGGCAGGTTTTTTAGAGCA | isolate patient no 3483 |
| TTTCTATTTCCACTGGTATTAC TCTATTGATGGTTTTTCCCACA | isolate patient no 2848 |
| CCCCATCTCTACTAAAAATACA gtcaGTACAGTTTATGTGCTTG | isolate patient no 2664 |
| AAATAAATAAAAATTAAAACAA ctctATAGCAGAGTAAAGTTAT | isolate patient no 1953 |
| CAATATGAATTGAACAACTAGG ggaTAGTCAAGTAATCACTCAG | isolate patient no 3045 |
| GGTAAAGGTGTTCAGTGATCAT ggGTCACTGTGCGAGGTCGCTG | isolate patient no 1449 |
| AATGCTACTACCAAAGGTGTTG CCAAAAGCTGGGATTACAGGTG | isolate patient no 1453 |
| AACATTCCTATCCATCCTGAGC GTGACCTTGGCCACTGCAATCT | isolate patient no 1699 |
| TATTTTCCATCCAAAGTTGTGT GCCTCTTTTTTTTTTTTTTTTT | isolate patient no 1829 |
| AGAATGCAGGCACTTTGAACAT CCTGACGTTCTGATGTTACTGT | isolate patient no 1912 |
| TTATTATCTGTTGCAAATGTGA GAATGCAAGAATATTGGGGGTT | isolate patient no 3064 |
| AGCTTGGGTGACACCGAGAacc CTGTAGTAGGTTCTTGATGATA | isolate patient no 2957 |
| TGTGAAGGCAAATAGGGTGTGA CTAGATAGGCTTTTTATTGAAA | isolate patient no 1235 |
| AGTTTAGGCTTTAGCCTGTTTC CCACAGGGAAAGGTTTTTTGTT | isolate patient no 1245 |
| GCACGGTGGCTCACGCTGGTAA AGGCAGGTTTTAATAGGAACTC | isolate patient no 1287 |
| TTGCACTCTAGCCTGGACAACA ttTGTTGGGGTTGTAGTTAATG | isolate patient no 1300 |
| GGAGGCTGCAGTGAGCCGAGAT TGGAAGTCTGATGTAGTTTGTG | isolate patient no 1328 |
| CTGGGCAGCACAGTGACACACA cAAACCTTTATATCGATGTCTG | isolate patient no 1459 |
| AGGAGGATCACGAGCCCACAAG CAACGGTGTACTTGAGAGAGCA | isolate patient no 1475 |
| GACCAGCCTGGGCAACATAGCA TTCTCATTTGAAGAAATATTTA | isolate patient no 1533 |
| TTTGCATTATTATCTGTTGCAA GAAAAGCTAATAGGCGAGGACG | isolate patient no 1534 |
| AAGGCTGTAGTGAGCTATGATT agGTGTAGGCTGCAGTGAGCTG | isolate patient no 1593 |
| CCTTCCCTGTATTCACTATTTT ACCTTCTCGAGGCCGTTATAAG | isolate patient no 1687 |
| TAAGTTAATTGTAATTAAAAGA TTTGTCATTTGCATTATTATCT | isolate patient no 1815 |
| GTAATTATTTTTTGACCCCAAC tTTGGACTTAGCTCCTTACTTT | isolate patient no 1834 |
| AGTTCAAGACCAGCCTGGGCAA GCCCACGGCTTATGTCCGGCCC | patient UPN054 |
| AAATAAAAATTAAAACAATTAA TCTTTTCTCATTCTGCAGCCTT | patient UPN047 |
| AGATCACTTGAGGTCAGGAGTT TACACATAAATTAGAACCAAGA | patient UPN044 |
| TCTTGACTTCTGcTCCTATAAC ACCTTGATTGTCTTCTACAAAC | patient UPN041 |
| TATCTATCTTTAAAATAGGTAT TTAACAGTTAAAGTTGGTTTTG | patient UPN036 |
| ATGAATTGAACAACTAGGTGAG CCCAAGAAATATCTTTGTGTTT | patient UPN030 |
| CATAGCAAGACCCTGTCTTaag ACCTAGATATCATCTACTTGTG | patient UPN029 |
| TATATTTATTTTGTTACTTTCT TTTTTTAAAAATTTATTCTTCT | patient UPN023 |
| ATATTAAGAGTGTGGTTGGATT GAGGCTTTTGTGCCATGGTAAG | patient UPN019 |
| TCTTGACTTCTGcTCCTATAAC ACCTTGATTGTCTTCTACAAAC | patient UPN017 |
| AATTACAAATGGAAAGGACAAA CCATTTTGCCTTTAATTTCCAG | patient UPN014 |
| ATATCAATATGAAAATAACTTA AATACCTCATCGTGGACTCCAA | patient UPN011 |
| AAAAAAGTGGCTCCCCGCCCAA TGTCTTTCACGGGGTTGCTCAT | patient UPN009 |
| AGTGTTCATTTCAATATTGCTC TTAGCAAAATGGGTATTTTTGA | patient UPN006 |
| CCATAGGGCATGATGCTTCTGA TTCTATCTTCCCATGTCTTACT | Homo sapiens isolate 4 |
| TTTTACCTGTAGATACACATGT TACAAAGtTTGTTTTTTTCTTC | Homo sapiens isolate 10 |
| CACTCCAGCTTGGGTGACACCG TGAATGACCAATCTGAGGCCAG | Homo sapiens isolate 7 |
| TTCTCTTACAGTTTTGCTGACT AGTTTCTCcTATGGCTTTAGAC | Homo sapiens isolate patient 68 (FT) |
| TGGAAAGTATCAAACCATGATG CAACCTGAAGCTTTAGTAGGAA | Homo sapiens isolate 16 |
| GATCACTTGAGGGCAGGAGCTG TTTAAAAGATGGTGGGTGCTTC | Homo sapiens isolate 15 |
| TGGCCTTTAGAATAAGCAGATT ttCATTACACATGCAAACAACC | Homo sapiens isolate 14 |
| GTATTCAAAGGTGGTAAAGAAA GTATTACCCAGTCTTTTCTGCT | Homo sapiens isolate 13 |
| CCACAGAAAAAaTAATTTATGT CCTGCAGCAAGGACCGATTTCT | Homo sapiens isolate 11 |
| GTCAACCCTGCCCACTTGCCAT GACTGTTATGGGATAAATAACa | Homo sapiens isolate 9 |
| CCAGAAAAAAAAATAGGCAAAA ATAGGTAATAGGTCAGAATTTA | Homo sapiens isolate 8 |
| ATAAGAAGGGTATGGTTGATTA GGATTTAGTGGGgTTTTTTTAG | Homo sapiens isolate 6 |
| ACAGTGACACACAGTTGCTATA GAATAGATATTTTATGCCCTAA | Homo sapiens isolate 5 |
| CTACATATTATTTGACATACTT ATAGATGGGTAGTTGCCCATAG | Homo sapiens isolate 4 |
| AATGCAGGCACTTTGAACATCC ATATCAAACAAACCTAGGGTTT | Homo sapiens isolate 2 |
| AAATAGGCAAAAGACAGAAATA TCTAGCTTTTCAGGTTCCGTTT | Homo sapiens isolate 1 |
| ATAACACCCAGGGTGGTTTGCT GTTTTGACTCAGAACCATCCGT | Homo sapiens isolate patient 020224 |
| AATTACAAATTATTGCCTACTA ATCCTTTTAATACTAAAAGCTC | Homo sapiens isolate patient 010922 |
| AACTTTTTTTATAGCACCAGca GACTTGTGTGTTTCTGTTACAG | Homo sapiens isolate patient 010814 |
| ATCCAAAGTTGTGTAATTGTAA GAATTTTAAAACTTATCTTTTA | Homo sapiens isolate patient 010613 |
| AATTACAAATTATTGCCTACTA ATCCTTTTAATACTAAAAGCTC | Homo sapiens isolate patient 02017 |
| GAATCTTGACTTCTGTTCCTAg TACTTTGAAAATAAGTGGTTCT | Homo sapiens isolate patient 00072 |
| GTTGCCTAGGCTGCAATACAGT AGCTGGAACTACTTTTAATTTT | Homo sapiens isolate patient 75 (GV) |
| CCATGACATATCACTGAGTGAA CTGTTGGACATTGAGATGGCTT | Homo sapiens isolate patient 74 (HA) |
| GATCACTTGAGGGCAGGAGCTG TTTAAAAGATGGTGGGTGCTTC | Homo sapiens isolate patient 69 (SA) |
| CCACTGCACTCCAGCTTGGGTG ccCTCCCTCAAACCCCTTTATA | patient UPN059 |
| CTCTCTCCACAGGAGGATTGTG GAGAGACAGAGTAGAGTGCTCG | patient UPN056 |
| TTCCTTCACATGGAAAGTAccg TAATTACTACTTTCTTGTTCCC | patient UPN055 |
| GCAGCACAGTGACACACAGTTG TCAGAATTAAAGTTTAATTCTT | patient UPN053 |
| ATCCTGAGCAGTATCAGAGGAA AAAGACTGCATCTGAATTTTTT | patient UPN051 |
| AAAAAAAAGTAGCCGGGCACGG AAAGATAATTAAGTCTATCTAA | patient UPN046 |
| GGGGCGGAGCCTGCAGTGAGCC AATTTTTTGTGTATATATTTTT | patient UPN040 |
| TCTTTGTGGCCCCACATGTTga GTAGGAGGCAGTaCTAAAGGTT | patient UPN039 |
| CTTCTTTGTGGCCCCACATGTT gaGTAGGAGGCAGTaCTAAAGG | patient UPN035 |
| CTCTCTCCACAGGAGGATTGTG GAGAGACAGAGTAGAGTGCTCG | patient UPN034 |
| ATATTCATCTTTTGTCTCCTTA AGTAAAAGAAAATTTATGCTGT | patient UPN028 |
| CTGTTAAATCTTGTATTATATc CAAACACATCATGTATTCAGCA | patient UPN027 |
| TTAATAGTCCGTGTCTGAGATT GATTTTCATAAAGCTTTATAAT | patient UPN026 |
| TCTCCTTAGGAAAAACCACCTC AAAATACAAAAATGAGCCGGGC | patient UPN024 |
| TATTATATTTATTTTGTTACct TGTTTCCATTTTTCTAGATATT | patient UPN021 |
| CCTATATCAATATGAAAATAAC AGTGGATTTTAGGAAAGGGGTT | patient UPN015 |
| AAAATTAGCCCGGCGAGGTGGC ATCTCTGTAAAAGAAGGGGATT | patient UPN013 |
| TAATATATAAAGCACAATCCCA GTTACCAGAAAGTTTCAGGAAG | patient UPN008 |
| CTGTAGTGAGCTATGATTGTAC ATCCCCTGAACTGAAACCACTG | patient UPN007 |
| GCAAATATTCTCTTAGTCCCTA AATTTGCATCTCCATTGCTCAA | patient UPN003 |
| AGCCTGGGTGACAAAGCAAAAC GAGTCCACAGTCTTAACCACAA | patient UPN001 |
| CTGCCTGCACTGCcACTCCaag TTTATGGGAGTATcCTAGTTCT | patient Pi |
| TTAATAGTCCGTGTCTGAGATT GATTTTCATAAAGCTTTATAAT | patient Ku |
| TTCAAGACCAGCCTGAGCACCA ACAGTCATCCACCAGTACCATC | patient Am |
| AAAATTTAGGCTTGGCAAGGCG cCAGGAAGCTCTCTTTGGTGCA | Homo sapiens cell line MV4-11 |
| GTATATTGAGTGTCAAAGACTT CTGCTCCCCTTTTGGAAAACCT | cell-line RS4;11 |
| GTCAAAGACTTTAAATAAAGAA GTGACCTCAGTTAAGTTATAGG | patient 15513 |
| TCTATATTCATCTTTTGTCTCC AATTTTTGACAGTAGTAGTATC | patient 12746 |
| TGATCTTTCTGTTAGATGTCTA AGCAGCCTGAGGGCTAGGTTGG | patient 6510 |
| CTGTAGTCCCAGCTACTCAGGA AGGGCTCTGTGATTGACAGGAG | patient 6343 |
| GATCATAAAGTATATTGAGTGT TTCACCATGTTGACCAGGCTGG | patient 6338 |
| TTGTTCTATATTCATCTTTTGT TAAAAATAAAAATTCCCAGGCC | patient 6238 |
| TTTTTATTTATTATGGATAgaa TTCACATGTGGCAGGCAGTTTG | patient 4085 |
| GAGCCCAGGAATTCAAGGCTGT ggAAAAAAAAAAAAAAAAAAAA | patient 4070 |
| ATTATATTTATTTTGTTACTTc CAAAGGCACAGTGTTTTAAAGT | patient 4062 |
| TCCAGCAGATGGAGTCCACAGG GCCTGCTGGATGTGTTTTTAGT | patient 4060 |
| ACAACTAGGTGAGCCTTTTtat ACTCAGATCCATGTATAAGAAG | patient 3954 |
| AATTCCAGCAGATGGAGTCCAC TTTGTAAAGTCACATGGGACCC | patient 3948 |
| GACAGAAATAGATAATTCACAA TTCTCTTCCCCCTTCTCTTCTT | patient 3940 |
| GCCTGGGTGACAAAGCAAAACA AAGGCAAGGATGGTGGGATGAT | patient 3934 |
| TTTGCAGTATGTACCACCTTTg AAAGCACTAAGCTCTGTAAAAA | patient 3885 |
| ACATCTATAATCCCAGCACTTT AGGGAAAAAATTATCAAAAGGT | patient 3676 |
| GATCGCGCCACTGCACTCCAGC ATGAAAAGCCTAGTGAAAGAAA | patient 3585 |
| AAGCAGAAAATGTGTGGGAGAT ATTTTATGTGTCTTAATGTGGT | patient 3548 |
| CCAGTCTCTTTTAAAAAAAAAT ggtgAGGAAAGGTGTAAGATTA | patient 3409 |
| TGGGAGAGCTTTGGTCAGTGTT GGATTTTCCAATACGTTCTCTC | patient 3361 |
| CAAAGCAAGACCCAGTCTCTTT aagGACCAAATCTGTTTATTGA | patient 3301 |
| GATTGCTTGAGGCCAGCAGTTC GTGGACTGTATTGGATGAATTC | patient 3256 |
| GGGGGAATGAATAAGAACTCCC TTTACTGAAATAATGGTGAGAG | patient 3224 |
| TTCAAGGCTGTAGTGAGatggg GTTGGAAACAACGGACAAATCT | patient 5457 |
| CTTAAAATTAAGAAACTTCAAG CAGCAGCAATAATCATTATTTG | patient 5336 |
| ATTATGGATAAAGAGATAGTGg GATGGAGTTTTGCTCTGTCTCT | patient 5301 |
| TAGTGGGTGCTTGTAATCCTAG AGTGGGAGGATGGAACTGAGGG | patient 5283 |
| TCTAAGCAAAAAATTCCAGCAG TTGGAACTCAGTTCCTGTAGAA | patient 5253 |
| TACTTTCTATTTCCACTGGTAT GACTACTGCTGCTCCTGCTAAT | patient 5208 |
| CAGTGAGACACAAACTAGCTAA AAAAAATTAAATTCTTCCGAAA | patient 5162 |
| AGTATCAGAGGAAGTAATaacc AGGTTCTATCTACAAAAACTCA | patient 5036 |
| GGAGGCTGCAGTGAGCCGAGAT GGCTTAACTATTTTGCTTGTGT | patient 4862 |
| ATAATAAAGAAACAGAAAagag CATATAAGAGCTGCTGCCACCA | patient 5722 |
| ACCTGTAGTCCCAGCTACTCTT TTTTGGGTTTGGGGCAGAAGGG | patient 6213 |
| ATGCTTTTCATCCTTATTTTCC TGGATAACCTTCAAAATGTAGA | patient 6165 |
| AAGAGAAATTCAATCCCAGTGT GTTTTTTAAAAATAACTTTGTT | patient 5995 |
| GCGCTGGGAGAGCTTTGGTCAG GGGTTGTAGTTAATGGTCTCCA | patient 5953 |
| GAGCCTGCAGTGAGCCGAGAga TTTATTATTTTAGAAGGACTTG | patient 5892 |
| GAAGCAGGCAGATCACTTGAGG GTATAATCAAAATGACCAACTG | patient 5829 |
| AAGTTTAGGCTTTTAGCTGGGC CACACTCCCTACATTTTCTTGA | patient 5733 |
| GGAGAATGCAGGCACTTTcctc TTACCAATATGAGCTGACATAT | patient 5691 |
| TAGGCTTTAGCCTGTTTCTTTT cggcAAAACCCCTTATATTTAA | patient 5679 |
| TTGCAAATGTGAAGGCAAATAG TTTTATGGGTAGTATTCTAGTT | patient 5625 |
| ATCCCAGTGTATTTTCGCAATA ATCTTTCTCATTTGAAGAAATA | patient 5458 |
| GAAAAGATCAATAATCCCATCT ATAGAGATCAGCCTACACTTAT | patient 5433 |
| ACCCAGGGTGGTTTGCTTTCTC ATTGCAGCCTGCTCTGTGCCTT | patient 5393 |
| GGTTTGCTTTCTCTGTGCCAGT ttTCATTCAACAAACTGGTATT | patient 5379 |
| AAGCAGCAGTTATTTTTGGACT TTGTTGTTTCTCATAAGAAACT | patient 5222 |
| ACTGACTGCAGAACATACATAA GGGCAGaGAGCCCTCATGACCT | patient 5187 |
| AATTCTGGATTATCCTAGTTAG TCTAGCTTTTCAGGTTCCGTTT | patient 3881 |
|  |  |
| AML1-ETO |  |
| AAAGACAGGGTAATCTGCGGCA AGGAAACgGGTTTCTTGAaAAA |  |
| AGCAGTGGGGACCCTAACTGAC ACGCTTTGTGGCCTtTGCCTCa | Homo sapiens RUNX1/RUNX1T1 |
| GATATTTGAGAGTTTTTTGTTT TTCTCCATTCACTTGCATAAAT | Homo sapiens isolate patient 26 AML-12-GLA-026 |
| AGAGCGAAAGGATTTTTCAAGA TTGTCATTCATAGTCCTCATTA | Homo sapiens isolate patient 24 |
| GCTGGTACACCCTCCGGCTGGT ttTTTTTGTTGCTGTGTAAAAT | Homo sapiens isolate patient 23 GM |
| ATAGAAAATCTATTATTGTTTT CTTCTaAGTGCCTGTTCTATTG | Homo sapiens isolate patient 22 KC |
| CTGGTACACCCTCCAGGCTGGT ttTTTTTGTTGCTGTGTAAAAT | Homo sapiens isolate patient 20 |
| TGAATAGCCAAATATTCTGGAT ATTTTAACATTGGAATCTTCAA | Homo sapiens isolate patient 19 |
| TAACTGCTGCATTTGTGGAACG TGTGCGGAGAGTGTGTGTGGGT | Homo sapiens isolate patient 18 GA |
| GATACAGGAAACTGAAGACATA cGGAGAATGATATCTGTCAAAA | Homo sapiens isolate patient 16 RT |
| CACCATTACATTGAATTTCTCT TATGCAAGGTAGAGGAAAGTAA | Homo sapiens isolate patient 14 LS |
| CCCATATCGGAAACATAGACTA TATAATGTGAGAAAAATGTGGA | Homo sapiens isolate patient 13 |
| CCTAATGTTTCCGCGAATTcct AACTCAAGAAGTTGGGAAACAG | Homo sapiens isolate patient 12 |
| TTGTGGATGCTTTTAGAGcccc GCATTCATCATGTTATTGATTG | Homo sapiens isolate patient 11 OP |
| TAAATGTTTCCAGCGAATTcct AAACTCAAGAAGTTGGGAAAAC | Homo sapiens isolate patient 9 FM |
| TGTTGTCTTAaCTTAACGATAT ACAAAGTCTTATAAAAATGTTT | Homo sapiens isolate patient 8 BS |
| TAgATATAATTAGATAATTAAA CATTAGGACCATGTGTAGCTTT | Homo sapiens isolate patient 7 SV |
| GGCCAGGTCCCCCGCTTGacct CTTGAATCCAAACACAGCTCAG | Homo sapiens isolate patient 5 WS |
| GCCCTCTTCCTGTAACTGccta CCTGCTAAAGTCTTGCTAGAGA | Homo sapiens isolate patient 4 GD |
| ATAGCCATGAGCCGAATAAACT AGTGCCTATGTAGGAATCTACC | Homo sapiens isolate patient 2 |
|  |  |
| ETV6-RUNX1 |  |
| TATTTACCATATGGAAAAACTA gtTCAGTtTAAAaAGATCTGGG | patient 382 |
| CAGAAGGTGAAGGGTGACCCAG CCAATGTGCTTTTATGCTTCCA | patient 480 |
| TTTCAGTGTGAACAACTGAGAA TTCAGGCCCCAGAAATGGGGTA | patient 463 |
| TTCAGACCAACCAGAATAGTTA GTatTTGCTAAAATGTATCAGC | patient 453 |
| TGATTTTCAGTGTGAACAACTG TCCCTAAACCCCTCCTCTAGCT | patient 469 |
| AAGAACATAAAATGACTTTCTT TTTCTGATCCAATTTCTCAGAA | patient 471 |
| CAAAGATTGAAAACTGTGGCCC TTTTGCCTGGTAGAGAAAGGCT | patient 429 |
| CCGTGGCTGCTGTCCATAGTGC CCAGTTAGGAATCAGCAATGCT | patient 420 |
| TTCCTCCCACAGTGTTTAAaTG CTTCTTTTCCTGCTTCACAATC | patient 407 |
| TGCACCACCAGTGTTGTCTCAG AAGCAATTAAAAGTAGCCCTAC | patient 388 |
| CGTTCAGGTGTTTTGAAGTGGA TACAGGTAAGTCATCTATAGTC | patient A22GM |
| ATGATTGGAATCCGGTTCTTGA GTTGAGCCATTACTGCAATCCA | patient A16DD |
| GGgAGAAATtGGTCTtGGTTCA TAGAAGAAAGGTCAATGGCTCT | patient A15GN |
| ACTGACATGGCTTGAATGTCAA CTCACATATGTGTTGTGATTCA | patient A13GP |
| GCTCTGCCAGTCTGTAGTCTGA AGGTCAGAAGTTCGAGACCAGC | patient A11EK |
| GAGTCACCAAGCCACATAGTTT GAATCAGAAATACATAGAATAT | patient 6581 |
| GGCCTTCTTTAAAATTCCctta AACACAACTTCGTGTCAGTGTA | patient 6954 |
| GAATACCTTGTGATCCAtAAAA TACAAAACACCAGCATTTGCAT | patient 6810 |
| CAAAGCGGAGGCTGGGCCTTAT ATAtATAGTAATAATGTAACCT | patient 6542 |
| CCTTGTAAATATACacccttcc AGCTACACACCCAACTGCTCTT | patient 6487 |
| AGTATGTATTGAAATTCCTTAT GTCTTGTTTGTGAGAGGAATTC | patient 6422 |
| CTACTTTACTTCTGaCTTGAGG GATTCTGCTCCCCTTTGTTGAA | patient 6367 |
| AGCAGATCTCTATTTTCCCTTT TTCAATGTGTGTTCATCATTTT | patient 6273 |
| CTGGCTTtTTTTCCCCCTAGAG GAGATTATATTCTaGGGCATCC | patient 6091 |
|  |  |
| MLL-AF9 |  |
| TTCAAAGTTCTGCCTAGATCTT ATCCTTCTATCTCAGCCTCCTG | Homo sapiens isolate 6 |
| tgGCCAAGTCcgGTTGTGAGCC GCTTTCACATGGgACcTTTTGG | Homo sapiens isolate 5 |
| TTGTTGGTATTTAGCAGGTACT CCTGGATTGTTCTTCTTAGTCT | Homo sapiens isolate 4 |
| TATGTTGACATGATTTCAGACT CTTGCAGAGGGTTACAGGGCTT | Homo sapiens isolate 2 |
| TTACCTCTAAATATTTTAGTGT CTTGTAACAAGTCACTTCCTCC | Homo sapiens isolate 1 |
| TTACTTTCTATTTCCACTGGTA GTATATATTACCTTATTAAAAT | Homo sapiens isolate U.G.(10) |
| TTTCTATTTCCACTGGTATTAC GGCTTTATTGTTGTTGCTATTG | Homo sapiens isolate F.L.(9) |
| CTTTCTATTTCCACTGGTATTA CCCATTGTTTTGGTAGTAAAGA | Homo sapiens isolate P.J.(8) |
| TAAATTCAAAGATTATTTGTTT TTAGTCGTATTTGAATGTGTGT | Homo sapiens isolate S.J.(7) |
| TTTCTATTTCCACTGGTATTAC GATAGGTAATTAGAATTCTGCT | Homo sapiens isolate H.D.(6) |
| CTTTCTATTTCCACTGGTATTA ATTGGAGGTCACAGTAAGCAAG | Homo sapiens isolate S.O.(4) |
| AGTTTGTCATTTATAAGGGACA GTATATATTACCTTATTAAAAT | Homo sapiens isolate V.C.(3) |
| CAAATGTGAAGGCAAATAGGGT CTTGTTACTCTTAGTTTTCTAT | Homo sapiens isolate O.M.(2) |
| CATATACACATTTTACCTGTAG CTCATCTCTCAGATTTTATAAA | Homo sapiens isolate R.L.(1) |
| TGATTGTACCACTGCAGTCCAG CCTCCGCCCTGTGAGGCTCGGA | Homo sapiens t(9;11)(p22;q23) translocation breakpoint junction |
| TGTTCTAGCCTAGGAATCTGCT GAAAGAAGGACCAAAACTGTGA | cell-line THP-1 |
|  |  |
| MLL-ENL |  |
| GGTATTTAGCAGGTACTATTCC TCATCCACCCACCCTTTAATTC | isolate patient no 4606 |
| ATCTTCCCATGTTCTTACTATA CCGAACTTCGCATCTGTTGGCC | isolate patient no 4562 |
| ctcGGCTGAGATAGAAGGATTG CCTGAGCTGCCTTGTATTTCAG | isolate patient no 4841 |
| TGTGAAGGCAAATAGGGTGTGA CTTTCAGGGTGGCCTGCTTTTC | Homo sapiens isolate 12 |
| AGCATGCTGCCTGCACTGCACT GTGAGCCACTGCACCTGGCCAT | Homo sapiens isolate 11 |
| AGGGCAGGAGCTGGAGACCAGC ATTTCCAGGTGGTCTACACACC | Homo sapiens isolate 9 |
| TATACAGTGGTCTATAAAAGGG CAAGTGACTTGCCTAAGGCCAC | Homo sapiens isolate 8 |
| CAGTCTACAAGTGCCAGGGGTC CTTCATCTAACGCCCATCAGCC | Homo sapiens isolate 7 |
| TCCAGAAAAAAAAATAGGCAAA GTAGCTGGGACTACAGGTGCAC | Homo sapiens isolate 6 |
| TTGTACATAGCAATCTCACAGG CTGTTGGCTCCAGGGGCATGGG | Homo sapiens isolate 5 |
| AGGATTGCTTGAGGCCAacaat TTCCTGTTTACTTTGGCCACTG | Homo sapiens isolate 4 |
| GAGGCCGAGGCAGGCAGATCAC CGTGTAAGCATAGAGGGTGGTC | Homo sapiens isolate 3 |
| GGAGAGCTTTGGTCAGTGTTGT ACTTTCCTTATATACTTTGCAT | Homo sapiens isolate 2 |
| TCAGTGTTGTTAGGTCACTGTT CGAGACCAGTCTGGCCAACATG | Homo sapiens isolate 1 |
| TGTCTTTATTTAAACAAAAtcc TTTGCCCATCCAGTAATTTATC | (MLL-MLLT1 fusion gene), patient 3321 |
| TACTTTCTATTTCCACTGGTAT CTCCACCCATCCCCTACCTGGA | (MLL-MLLT1 fusion gene), patient 5127 |
| CAGCTTGGGTGACACCGAGAtT CGAGCGTCTCAGACTTGAGGAA | (MLL-MLLT1 fusion gene), patient 5064 |
| GGAAGGAATCTGCTATTAGAGT TGGACCCAAAGTCTGATTCTAG | (MLL-MLLT1 fusion gene), patient 4938 |
| TTAAATAAAGAAAATGCTACTA GGAACTCCCTGTCATgAGAAAT | (MLL-MLLT1 fusion gene), patient 6104 |
| TTTGAAAGCAGGAAATGTATGA AAGATTGTTGCAAAGAGTTAAT | (MLL-MLLT1 fusion gene), patient 6320 |
|  |  |
| BCR-ABL |  |
| GGAATGGGGTTGGGAGAGAGGACTAACTGCAGATGAACCCAAGGGGGACTTTTT TGTCTAAACAAGTTAATCATGCACAGATCCAGTGAATTCT | patient 6359 |
| CTTGTTAGGGCCTCTTGTCTCC AGTCTCTGTGTGTCACCCAGGC | patient 6904 |
| AGAAAGTTACAACCTTTTTTTT GGACCTATAAAACTAATGTAGTT | patient 6855 |
| GGTTTGCCTGTATTGTGAAACCA CAGGATACACAGTAGGGTGATTGGTTT | patient 6853 |
| GCTGATCCCCCCTTCCTGTTAG ATTATATGTATATGCCCACAAA | patient 6832 |
| GATGAGTATGTTTTTGGCCCAT TTAGTAGAGACAGAGTTTAGCC | patient 6826 |
| TCTCCCCTAGCCTGTCTCAGA AAGTGGGAGAATCACCTGCGCC | patient 6815 |
| TGGTTGATGCCTTCTGGGTGTG CAGTCTCACTCTGTTGACCAGG | patient 6794 |
| CTCTGTCGAGCTGGATGGATAC CACCTGAGGTCGGGAGTTCAGG | patient 6720 |
| CGGGACACCTTTGACCCTGGCC CGGATATCTAATACCACTTTTA | patient 6658 |
| TTGCACTGTGTAAGTTTCTCGA AGTTCAAGACCAGCCTGGACAA | patient 6651 |
| ATTACATGACATGCAGATTGCA AACTAGTTGGGGCATCTTTTTT | patient 6568 |
| GCCACTGCATTCCAGCCTGGGC TTATGCAGGCTGGAGTGCAGTG | patient 6563 |
| TAACTCTTTGCCCCATAGTACA TTTGGAGATAGGGTCTTGCTCT | patient 6552 |
| TTGTGCCAGGCAGATGGCAGCC ggaCACTGCCTGTCATCTTCAC | patient 6541 |
| ACCTATTATTCATGGACCCCAA AGGTGGGTATGAAATTGATCTG | patient 6521 |
| ACTGGTTTGCCTGTATTGTGAA CAGCACAAGGCAAGCAAATATC | patient 6516 |
| GGAAGGACTCATCGGGCAGGGT AAAATTAAAGGGAACGTTTGTC | patient 6513 |
| TTCCTAGTCACAAGGCTGCAGC TAGAAATGGGGGTGACATTCAC | patient 6433 |
| TTTGGGTATTTTGTGAATAAAG GAATTTCTACATCAAAAGAATA | patient 6400 |
| TCCGTGTACAGGGCACCTGCAG TTTCAAAAAGTTAATTTTTTC | patient 6385 |
| CCTCCCAGGAGTGGACAAGGTG CAATGGTGCAATCTTAGCTGAC | patient 6379 |
| GCGCAGTGGCTCATGCCTGTAAT GCTGGAGTGCAGTGGTGTGATC | patient 6350 |
| CCGTGGTCCCGGGCTTGTCTCT GGTCTGTGTAAGAAATCTTTGT | patient 6340 |
| GCCCATGACACTGGCTTACCTT AGAGCGCCTGCTGTTTGATTTT | patient 6322 |
| TTCTGATTCTGCAAATAACACC GAAGGATAAAACTGTCTTTT | patient 6296 |
| GATGCTCTGTGCCTTGGATCTG CCGGAGACGCGGTCCGGCCTAC | patient 6283 |
| GACACTGGCTTACCTTGTGCCA CTCTGTTGCCCAGGCAGAGTGC | patient 6271 |
| CTCCTCAAATGCTCTGTGCCTT ACAGCCGCTCCCCATAGTACTC | patient 6264 |
| TGGGTTAGGAGCAGTTTCTCCC GGACAGGCAGCTGATGTATGGA | patient 6223 |
| CAATTAGGTGTTTAATTTTTAA CCACCCAGTCTTGATCATCTCA | patient 6208 |
| CAGCTAGCCTGAAGGCTGATCC CCTAAAAGTTTCCAAAGAGA | patient 6192 |
| GCAGACGCTCCTCAGATGCTCT TGATTGGTTTGGGAGGGCACTG | patient 6188 |
| ATGGCAGCCACACAGTGTCCAC TAATGAATAGCTGAACTTGCAA All | patient 6076 |
| TGAGATCCCCAAGACAGAAATC GTGAACTTGGCTATCACTTCCT | patient 5899 |
| ACGGCTTCTGTTCCTAGTCACA TTTGCCTTTAAAAATATATATA | patient 5873 |
| AACCAAACCTATTATTCATGGA GAACCAAAGTAATCCCACAGTA | patient 5822 |
| CTCTCCTCCAGCTACCTGCCAG CCTGGCCTATGCTTGCTGCCTT | patient 5810 |
| CCCATGACACTGGCTTACCTTG GGAAACTCGCCAAAAGCGGTGC | patient 5793 |
| AAGGCTGATCCCCCCTTCCTGT AAGGTTTAACAACTCTCTCACA | patient 5726 |
| AGTGGCTGCTGCTGGGTGGTTG TGGTGTCTGTTAGGAATCTGGA | patient 5699 |
| GCTACCTGCCAGCCGGCACTTT GAGAATGAGGTACTTTAAGATA | patient 5655 |
| GCCGGCACTTTTGGTCAAGCTG AGGAGTTTCCAGTAAATTAATA | patient 5626 |
| GCTTGTTAGGGCCTCTTGTCTC CACGCCACTGCACTGCAGCCTG | patient 5587 |
| AAATTCCACAGAGCGGGCAGGG TGGAGCCCTTGAATTCTATCCC | patient 5440 |
| AACTGCTTGGGAGGCTGAGGGA GGGGAGTTTATCCTTTCCTTCT | patient 5349 |
| GAGGGGCACCACCATCCACCCGC gtgtGAGAGGTGGGGGTGATCAC | patient 5217 |
| GCCGGCACTTTTGGTCAAGCTG CTTTTATCTGAGACAAAGTGGG | patient 5183 |
| CTGTGGAGTGTTTGTGCTGGTT TAGTCAAAACAAACAACTCCCA | patient 5171 |
| CTCTGTCGAGCTGGATGGATAC ATGGTGGCTCATGCCAGTAATC | patient 5097 |
| AAAAAAAAAAAAAAAGTTCCTA CATCCACCATATTCGCTCCAAA | patient 5066 |
| TTAGCACTTTTGATGGGACTAG TGGACTTCTAAGGCTTCCTGAA | patient 5063 |
| TTTCTGAATGTCATCGTCCACT AAGAAATAGTATTTTAGTATGG | patient 5045 |
| TCCCCAGTCTCAGGTAGTTTTT TCAAAAAAAATTTGTTAACTTT | patient 4972 |
| TCATCTCTGGCTGCCTGGCCAG ATATTTGCTGGTACCTCACCCC | patient 4930 |
| TATTACACTTCGAGTCACTGGT CCCCCAGGCTGGAGTACAGTGA | patient 4859 |
| CGTCACCCCGACCCCCTCTGCT AATATGAGGAAAGAgAGAGATA | patient 4836 |
| CCTCCCTGCATCCCTGCATCTC TCTTTCTTACTAGTTAAGCTAC | patient 4810 |
| GAGTATGTTTTTGGCCCATGAC TGTGGAGCTGTGCCCTTTCTGA | patient 4768 |
| AGTGGCTCATGCCTGTAATCCC TGCAAAAGATGTTAATATGTTA | patient 4658 |
| GGATTGTTGGGGAATGGGGTTG TTGTCCCCCTGATCATTACTGA | CML46 |
| CTCCAGCTACCTGCCAGCCGGC TTCTCTTATGTAACTATAGTAT | CML45 |
| CTGTTACCTTTCTTTCTATCTC GGCACTTCAGCTTTCTTTTGAT | CML44 |
| TGTGGGAAGTCCCGTTTCCCAG TTCGTTTTTGCGACAGAGTCTT | CML43 |
| GAGTTGGAGACCAGCCTGACCA cACCCTTCTACATCTTTCTGTC | CML42 |
| TTCCTCTTATGTCCTGTCCCTT ATTAAAAAATCAGTTGAGCCCA | CML41 |
| GCCAGAAACCGTGGTCTGCTCT TTTCCCCTTTTTTATTAGCAG | CML40 |
| AGGTGTTTAATTTTTAAAAAGA CTGAGAGACAAATACAAAAAGG | CML39 |
| GGGTCTCCACCCAGGAAGGACT AAACGAACTCTAGGGAGAGTAA | CML38 |
| AACCCTACACTTGGAATGGATG TAGGGTCTTTGTGAGAATTAAA | CML37 |
| AATCTTTCTCCTGGGCCCCTGT AGTAATCTCTTCCTCGTTGATG | CML36 |
| GGCCTCTCCATCCCCAGTCTCA AAAATACAAAAAAATTAGCAGG | CML35 |
| CCACAGAGCGGGCAGGGGCATC TTTGTAAACATTTTTCATCAGT | CML34 |
| CACACACCCCACCCACATCCCA TGTGTATCCTCAGTCTGATGGC | CML33 |
| GGGTGCCCGGGAGTGTGGGGTC AGGGTCCTGGAGGTTTGCCACT | CML32 |
| AGGTGAGAGCAGTGTCGTGAAA GGCTCAGGCAGTCCTCCCACCT | CML31 |
| TACTGGAGCTGTCAGAACAGTG TTTAGGTAATTTAATTTTTTTT | CML30 |
| GCAGATGGCAGCCACACAGTGT GTCTTTTTGTTGTTGGCTTTTG | CML29 |
| TGGTCAAGCTGTTTTGCcTTCA GCTGCGGATGGAAGTCACAAAA | CML28 |
| CTCTTACAGACCATGTGGGTGA AATTAGGAAGTGGGGCAAAAGC | CML27 |
| CATGACATGCAGATTGCACCTT GTTGAGAAAGGTCTGTCGATAG | CML26 |
| CGCATGAGGTGCTGGTGTTCAC TCAGTTCAGGACAGAACCGTGC | CML25 |
| ACTCTTCTCCAGGCCTCGCCTC ATAGACTAAGGAGCCACATCCC | CML24 |
| ACTCTTCTCCAGGCCTCGCCTC ATAGACTAAGGAGCCACATCCC | CML23 |
| GTCAGCAGTGCACCTTCACCCC TAGTTCATAAAGATGTATTCTG | CML22 |
| ACTGGTTTGGGGAGGAGGGTTG GAGGCATGGGGGTCCACACTGC | CML21 |
| TGGCCAAGCCAGAAACCGTGGT TAACCTAAAAAGCTGTGGTGAG | CML20 |
| AACCAAACCTATTATTCATGGA TCTTCATGGTTTATGTTCTGTT | CML19 |
| ACCTGCCTCCCTTTCCCGGGAC GTGAGCAGGGGAATCGAGGAAC | CML18 |
| CTGTGGCCTCCTCCCTGGTCTT AGGTAAATCAGATATTAAATTC | CML17 |
| GCAAACCCCACCCTGCAACTTA TCTATAGTCCCAGCTACTTGAG | CML16 |
| GGTGCTGTTTGCGCTCACATTT GGGTTACTGTTTGTACTGTTGC | CML15 |
| CGAGCTGGATGGATACTACTTT CAGACGTGAGCCACTGCGTCCG | CML14 |
| CTTTGGGAGGCTGAGGCAGGTG TTCCTCCTTTTGTGTCTCTGAG | CML13 |
| AATCACAACTGCTTGGGAGGCT TTTTTAATTAAAAAAAATAGAG | CML12 |
| TCTTTAAACCCTACACTTGGAA AGTTTGCAAATTTGTTAGGCCA | CML11 |
| TGTCGAGCTGGATGGATACTAC CCGCTTAACTTCCCAATTTGCT | CML10 |
| GGTGGTTGAGGAGATGCACGGC GTGAACCCAGGAGGCGGATCTC | CML09 |
| TCTGCAAATAACACCTGCTCTT TTGACAAGTAGTTAGGTTACTG | CML08 |
| ATGTGGGTGATGTGGAAAAGAC TGTGTATCGTATGTATGTTTAT | CML07 |
| GCACCTTCACCCCACAGCAGAG GTATAATTTACATATCATAATA | CML06 |
| CTGGATATCCCTGCAGAAAGGG AAAATAAATTCATAGGTTTGTT | CML05 |
| TCTTTGCCCCATAGTACAGCGG TTGACATGCGTTTGGATTTTCA | CML04 |
| TAACACCTGCTCTTACAGACCA cTGCTTCCTCCGTAGTTCACTC | CML03 |
| TGTTAGCACTTTTGATGGGACT TCCAGTAAATTAATAATGAATT | CML02 |
| GTCTGTGAGCAATACAGCGTGA AAATTAAGCAGTAAACCTAAAG | CML01 |
| ACTGCATTCCAGCCTGGGCGAC TACACGTCGAGTCTTACAGCCT | isolate 60 |
| AAAAAGTTCCTAGAAACAGCAA CAGAGCTAAACTTTAGAGTCCT | isolate 59 |
| GAGATCCCCAAGACAGAAATCA ggacTTATGGCTCACACAGCGT | isolate 58 |
| CTCTATGGGTTTCTGAATGTCA GGATATAAGGAGAAAACATTTT | isolate 56 |
| TGGTGTTTCCCTCGTGGGCCTC GGCTAAGTAAGGTGCCATGAGA | isolate 54 |
| CAGATGACCACGGGACACCTTT AATTTCCAAGTGTAATGAAAGC | isolate 53 |
| GTGCTGGTGTTCACGCCAGACC GCATTGAACAAGTACTAAGTGC | isolate 52 |
| TGTCTACTAAAAATACAAAGAT CAAAAGTGTGCATTTTAGAGTT | isolate 50 |
| TGTTACCTTTCTTTCTATCTCT CCTGACCTCGTGATCTGCCTGC | isolate 49 |
| TTTATTTTTATTTTTTCTGATT GGGTTCAAGCGATTCTCCTGCC | isolate 48 |
| CATCCCCAAACCAAACCTATTA CAGGGTCTCACTATTTTTACTA | isolate 47 |
| CCTGCAGAAAGGGTCCCCACTA ATAGAGAAGGAAAACTAAAACA | isolate 46 |
| GAAAAGACTGTGGTGCTGTTTG AAATGTACGTGCCAATGACCCA | isolate 45 |
| TGGAGACAGAAAGCTTACCAGG ATAAAACTGTCTTTTCTGGCCA | isolate 42 |
| AGCAGTTTCTCCCTGAGTGGCT TAGCAAGGAGGGATGAAATTGC | isolate 41 |
| TGGGTTAGGAGCAGTTTCTCCC cAAACTCCATTTCAAAAAAAAA | isolate 40 |
| TCTGAATGTCATCGTCCACTCA AAGTTCACTCAAGAAGTAACCA | isolate 39 |
| TGGGTGTGGAATTGTTTTTCCC CATGGTTTATGTTCTGTTGGTT | isolate 38 |
| TCCACTCAGCCACTGGATTTAA CTAGCCTTTTGAATTCAGGGTT | isolate 37 |
| CCATGTCCACTTCTCCCCACAG CCATTCAGAAAAATTACCAAAT | isolate 35 |
| ACCCAGGGAAATTCCACAGAGC ATTTTGTGAGCAAACAAATATG | isolate 34 |
| TTCCGTGTACAGGGCACCTGCA aATGTAGGAGCTGTTATCCCTT | isolate 33 |
| TCAGTCACACACACAGCATACG TATCCCAGAGAACTCCATATTG | isolate 32 |
| CAGCCTGGGCGACAGAGCAAGA TTTAATTGATTGCTTCTCTTTC | isolate 31 |
| GGTGCCCGGGAGTGTGGGGTCC cATAAAGGTAGAGTGGTGAGAA | isolate 30 |
| TCCCAGGAGTGGACAAGGTGGG GCCGGGCGCGGTGGCTGACGCC | isolate 29 |
| GAGCTTGTGCCACTGCATTCCA TGTTAATTGTGGAAAAAAATTT | isolate 28 |
| CCTAGCCTGTCTCAGATCCTGG TAGGCAGAGGTGGGAGGATCAC | isolate 25 |
| CAAACCCCACCCTGCAACTTAC AGAGTACTGGGGTGTCATGTTC | isolate 24 |
| CCCCGACCCCCTCTGCTGTCCT CACGCCGATAATCCCAGCACTT | isolate 23 |
| AGTCACTGGTTTGCCTGTATTG GAACATATTTGGCATTTGAATG | isolate 20 |
| CCACTCCCGTCCTCCCAGCCCT CCCCCAGCAATTTGGGAGGCTG | isolate 19 |
| TTTTAGGTGAGAGCAGTGTCGT AGCAGATAAATAAATGCCTTCA | isolate 18 |
| CCATGACACTGGCTTACCTTGT CCACCACCCTGAGTTAACTACT | isolate 17 |
| ACCCCGACCCCCTCTGCTGTCC GCCACTGAGTATTTCCTTCAAA | isolate 16 |
| GGGCTTCCCACATCCCCCAGGA CATACATTTTTCTCTATTCACT | isolate 15 |
| ATTATTCATGGACCCCAAACTT TGATTTTTATTTTTATTATTTA | isolate 14 |
| CTCCCAGCCCTCCTCTCCTCCA ATTTCACTTTCTTTTCCTTTTT | isolate 13 |
| AGAGCAAGACTCCGCCTCAAAA CAGCTCTTATTTAAAAAATCCT | isolate 12 |
| TTCTCCATCAGTGAGGCTTCTT CAGGAGGCCAAGGCGGGCAGAT | isolate 11 |
| CAGAGCGGGCAGGGGCATCGCA AAACAGAATTATATAAATCTCT | isolate 10 |
| TGTAATCACAACTGCTTGGGAG tCGAAAGGGCCATGGGAAAGAA | isolate 9 |
| TCCCGGGCTTGTCTCTCCTTGC AGTAGAGATGGGGTTTCGCCAT | isolate 8 |
| GAGTGGACAAGGTGGGTTAGGA AAACCTCATATCTTTTAATTTT | isolate 7 |
| CCCCGACCCCCTCTGCTGTCCT ACTGATCAGTGCACCCATGTGA | isolate 6 |
| ACATGTGTCCACACACACCCCA AAAGTGTTTTCCCGACAGTATC | isolate 4 |
|  |  |

| **Fusion types** | **Sample number** | **fsgRNA number** | **Low off-target fsgRNA**  **(%fsgRNA number)** | **Minimum off-target fsgRNAs**  **(%fsgRNA number)** | **Sample**  **with**  **fsgRNA**  **(%sample number)** | **Sample**  **with Low off-target fsgRNA**  **(%sample with fsgRNA)** | **Sample**  **with Minimum off-target fsgRNA**  **(%sample with fsgRNA)** |
| --- | --- | --- | --- | --- | --- | --- | --- |
| **BCR-ABL** | 153 | 430 | 191(44.2%) | 59(13.72%) | 133(86.9%) | 83(62.4%) | 40(30.1%) |
| **MLL-AF4** | 169 | 279 | 142(50.8%) | 30(10.75%) | 130(76.9%) | 90(69.23%) | 22(13.0%) |
| **MLL-AF9** | 16 | 23 | 9(39.1%) | 1(4.4%) | 12(75.0%) | 5(41.7%) | 1(8.0%) |
| **MLL-ENL** | 20 | 29 | 12(41.4%) | 3(10.3%) | 16(80.0%) | 8(50.0%) | 2(13.0%) |
| **EVT6-RUNX1** | 20 | 42 | 16(38.1%) | 4(9.5%) | 17(85.0%) | 13(76.4%) | 4(24.0%) |
| **AML1-ETO** | 20 | 39 | 15(38.5%) | 8(20.5%) | 15(75.0%) | 11(73.3%) | 8(53.3%) |
| **In total** | 398 | 842 | 385(45.7%) | 105(12.5%) | 323(81.2%) | 210(65.0%) | 77(23.8%) |

**Table S2.**The off-target analyses
